# Land, lava, and disaster create a social dilemma after the 2018 eruption of Kīlauea volcano

**DOI:** 10.1038/s41467-021-21455-2

**Published:** 2021-02-22

**Authors:** Bruce F. Houghton, Wendy A. Cockshell, Chris E. Gregg, Brett H. Walker, Karl Kim, Caroline M. Tisdale, Eric Yamashita

**Affiliations:** 1 Department of Earth Sciences, University of Hawai’i, Honolulu, HI USA; 2 National Disaster Preparedness Training Center, University of Hawai’i, Honolulu, HI USA; 3 Department of Geosciences, Eastern Tennessee State University, Johnson City, TN USA; 4 Department of Urban and Regional Planning, University of Hawai’i, Honolulu, HI USA

**Keywords:** Natural hazards, Volcanology

## Abstract

The unprecedented cost of the 2018 eruption in Hawai’i reflects an intersection of disparate physical and social phenomena: widely spaced, highly destructive eruptions, and atypically high population growth. These were linked and the former indirectly drove the latter with unavoidable consequences.

Kīlauea is one of the most active volcanoes on our planet^1^ and has one of the earliest, most comprehensive volcanic monitoring systems^2^. Some 90% of its surface has been re-paved by new lava in just the last 1100 years^3^. Its recent history has been dominated by activity at the summit caldera and from one (of two) lines of vents called the Eastern Rift Zone (ERZ). The interval 1967–2018 was dominated by eruptions from the upper part of the ERZ^3^. In contrast, no damaging eruptions occurred after 1961 in the more heavily populated Puna district from the vents within the lower portion of the ERZ (LERZ). Long-term recognition of the high threat to Puna from lava flows from the ERZ^4,5^, depicted on the 1974 USGS lava hazard map, ensured that land values were always depressed in lower Puna (within the three highest hazard lava zones 1–3). The capitalization of high risk into low land prices attracted people to this beautiful but hazardous area. Steep population growth occurred^6^ during the absence of any locally sourced eruptions between 1961 and 2018, and set the scene for the unprecedented levels of infrastructural damage during the 2018 LERZ eruption. Valuable lessons regarding complex interplay of science, policy, and public behavior emerge from this disaster.

## Kīlauea: the 2018 LERZ eruption

**Fig. 1:**
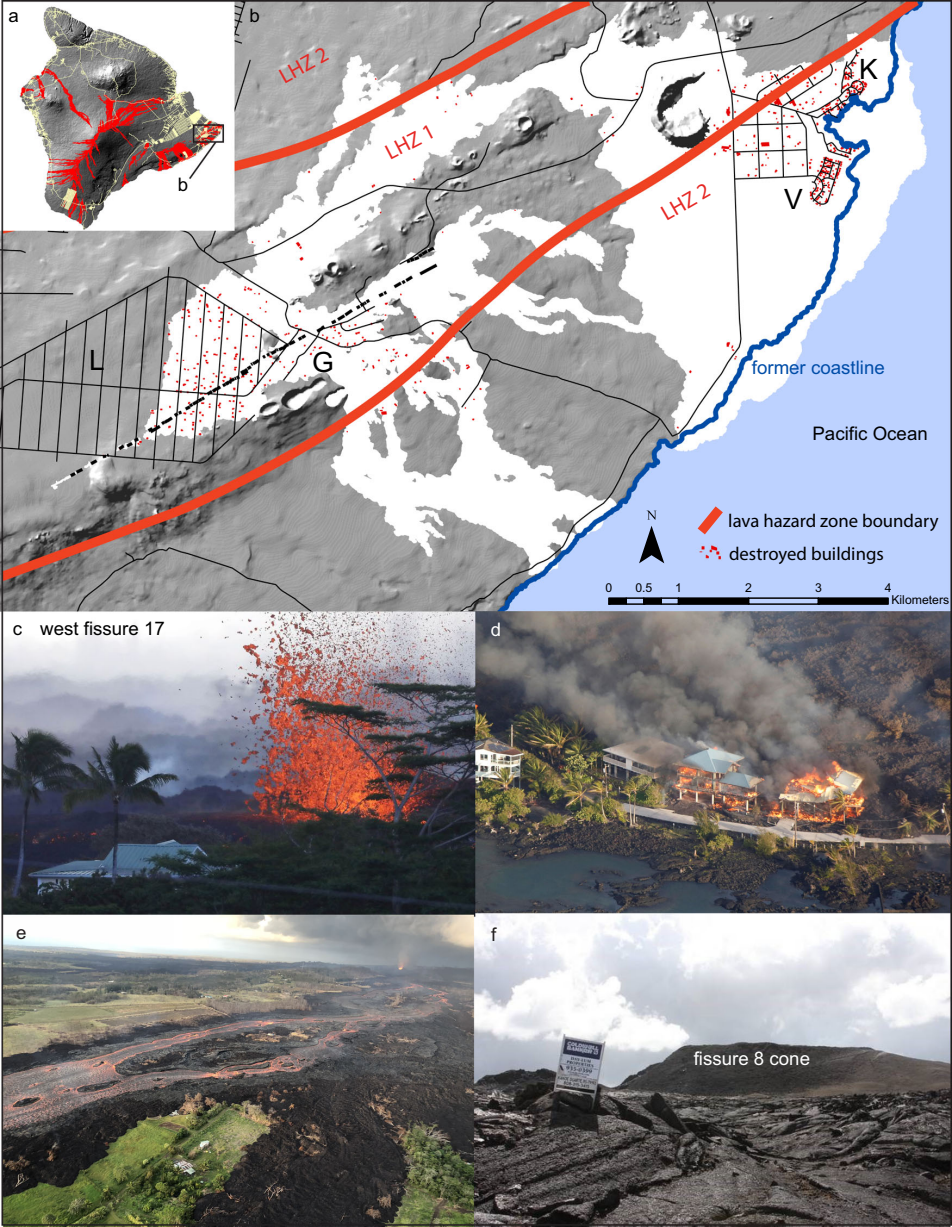
Images and impacts of the 2018 LERZ eruption. Much of Kīlauea volcano (and its neighbor Mauna Loa) have been re-surfaced by new lava in the very recent past. The areas colored red in **a** are lavas emplaced in the last 200 years. Lavas from the 2018 LERZ eruption impacted directly on only a tiny portion of the County of Hawai’i^7^, some 0.34% of its land area (white areas in **b**). Significant destruction in the Leilani Estates and Lanipuna subdivisions (**L** and **G** in **b**) occurred in May. Later activity spread further and mostly to the southeast. **b** From late May to July 2018, lava flowing first northeast then to the southeast, devastating the Kapoho Beach Lots and Vacationland subdivisions^8^ (**K** and **V** in **b**). Two forms of structural damage resulted. 1. Houses close to the fissures were bombarded by projectiles (**c**) and/or damaged by ground cracking and hot volcanic gas, especially SO_2_. The image shows a house that was damaged but not destroyed by large projectiles. 2. Houses in the direct path of the lava either were burned (**d**) or surrounded by the flows (**e**). The houses in **d** were located on the coast at Kapoho tide pools. Several large ‘islands’ like the one shown in the foreground in **e** were isolated and without access to County electrical or water supplies. (Note fissure 8 fountain in the background). The time required to return the lava-covered land to its former state is probably decades. **f** shows the margin of the lava flow field from the fissure 8. The main 28-m-high cone constructed during this phase of eruption is in the background. Flow thicknesses here range from 5 to 15 m. Closer to the former coastline, lava reached thicknesses of 55 to 280 m^7,8^. Images **c**, **d**, and **f** are courtesy of Getty Images (Photographer Mario Tama), Reuters (Photographer, Terray Sylvester), and the Hilo Tribune-Herald (Photographer, Tom Callis), respectively. Image **e** Photographer Bruce Houghton.

The 2018 summit and flank eruption of Kīlauea volcano was one of the largest volcanic events in Hawaiʻi in 200 years, erupting close to 1 km^3^ of new lava^7,8^, and ranks as the costliest volcanic disaster in the USA since the 1980 eruption of Mount St Helens. Major infrastructural damage occurred in and adjacent to the LERZ (Fig. 1). The eruption response could be considered a success in that no lives were lost. However, lava flows burnt and/or crushed most structures in their path; 723 buildings were destroyed, 3000 residents were evacuated and two major highways and a total of 50 km of public roads were cut by the flows^9^. The flows impacted four residential subdivisions in Puna (Fig. 1)^7,9^. Figure 1 illustrates that all the destroyed houses lay within the lava hazard zones 1 and 2. Locally, the boundary between no impact and total devastation was always exceptionally sharp, leaving some homes untouched (Fig. 1e), but isolated and without water and electricity. The total cost of recovery from the eruption has been estimated at greater than $US 800M^9^. Puna had suffered significant infrastructural damage in at least four previous eruptions including the destruction of the communities of Kapoho (1960), Royal Gardens (1983–2012), and Kalapana-Kaimū (1990–1991).

## Population growth on and beside the LERZ

**Fig. 2:**
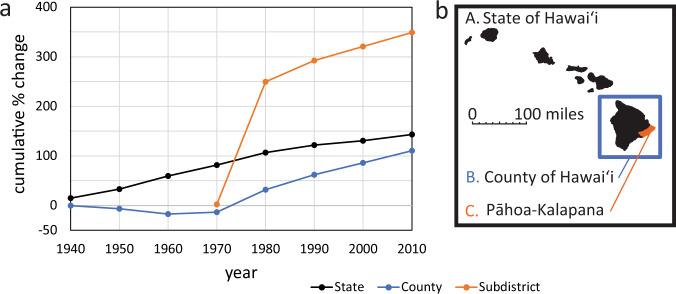
Percentage changes in population growth in Hawai’i. **a** This figure shows the data for the State of Hawai’i, the County of Hawai’i, and the Pāhoa-Kalapana census division. **b** The latter is centered on lava hazard zone 1 on Kīlauea’s LERZ. In Pāhoa-Kalapana, (where data is first available in 1970), a steep spike in population growth occurred from 1970 until 1980, which was unmatched in growth rates in the rest of the county or in the state. Subsequent growth (1980–2010) has been steadier at 5.7% per year respectively but still ahead of the remainder of the County and the State of Hawai’i. The exceptional growth in Pāhoa-Kalapana was largely driven by major land subdivision and sale in parts of Puna within the three most hazardous lava zones on the island. Some 80,000 new properties were created in the county between 1968 and 1975^6^, at a time when the population of the entire island was less than 80,000.

Unlike the state and county of Hawai’i as a whole, there was a steep peak in population growth between 1970 and 1980 in the high-risk area surrounding the LERZ (Fig. 2). This accompanied the subdivision of large blocks of land formerly zoned for agricultural use. In lower Puna, housing development within subdivisions in lava hazard zones 1 and 2, during a pause in LERZ eruptions from 1961 until 2018, created a growing population at risk from volcanic disasters^6^. In economic terms, living in Pāhoa-Kalapana still seems desirable in 2020 relative to the rest of the state, as seen in 2019 comparisons between Hilo and Puna counties in terms of median cost of land ($252,000 versus $28,500) and median household income ($52,563 versus $40,545)^10,11^. At the 2010 census, Puna had 24% of the county’s population on only 10% of the land area. Puna’s population grew by 66% between the last two census counts (compared with 24% for the entire island). The population contains many residents who commute from Puna to Hilo, or to the west coast of the island where land is scarcer, and prices much higher.

A link between rapid population growth and the cost of natural disasters is not unique to eruptions suggesting lessons learned at Kīlauea have much wider application. For example, the growth of coastal populations, in the United States and elsewhere, has greatly increased exposure to coastal flooding, hurricanes, and tsunamis^12^. What is more unusual is that the high volcanic risk at Kīlauea actively promotes rapid population growth, via the feedback into cheap land prices.

## The inevitable convergence

Kīlauea eruptions have always provided challenges for the planning and management of sustainable development. On short time scales, there has been no way to predict the timing and exact location of lava-producing eruptions from the LERZ (or the middle ERZ). However, on a scale of decades, potentially damaging eruptions are unavoidable. The interval 1967–2018 was dominated by eruptions from the middle and upper ERZ^3^ but no eruptions from the LERZ had occurred since 1961, the period of land subdivision followed by rapid growth in population^3,10^. However, the return of damaging volcanism here was inevitable on a longer time scale, given the recent history of damaging eruptions in 1955, 1960, and 1961 and the effects of MERZ eruptions in Puna in 1977, and between 1983 and 2015.

## Current and future challenges

Communities and land are altered for generations in lava-producing eruptions. A first-order unresolved question is what risk levels are acceptable at Kīlauea? There is, and always will be, a high level of long-term risk of lava inundation from Kīlauea’s eruptions, especially in hazard zones 1 and 2^4,5^. The willingness of people to move to affordable land in lava hazard zones 1 and 2 speaks to broader socio-economic problems that extend well beyond Puna district^6^. Data suggests that the repose interval in the LERZ is, on average, 14 years over the last 1500 years and 43 years for the last two hundred years^13^. Damaging hazard events with this intermediate frequency are inherently difficult to accommodate in land use planning. Events that are much more frequent than this require clear solutions; much more infrequent events can be largely discounted on human time scales. While nothing had changed in terms of eruption frequency on the LERZ, the steep population growth since 1970, thanks to low land prices driven partly by volcanic risk, meant that in 2018 more residents were vulnerable than ever before. Because of the large number of people still living on and adjacent to the LERZ, the hazards, threats, risks, and limited alternatives, present in 2018, persist today. People live here for a range of reasons that includes: long family attachment, the desire to be off-the grid (physically and mentally), the feeling of living on a living evolving landscape, and the depressed prices. Residents are thus likely to have different criteria and levels of tolerance with respect to risk from the volcano.

The county of Hawai’i is now restoring the major public roads affected by the eruption. This strategy, in essence, will restore the status quo for road access into the affected parts of hazard zones 1 and 2 and permit residents to return to many currently isolated properties (Fig. 1e). Beyond this, the repair of roadways may inadvertently encourage continued unplanned urbanization and increased development and investment in hazard zones 1 and 2 with the negative outcome that it does not reduce but rather perpetuate risk. The only alternate long-term option that can improve the risk landscape in and adjacent to the LERZ is by choosing different land use patterns^14^. However, such a strategy is probably not viable in the current social and economic climate? Decisions made today will affect the long-term safety of future generations in Puna.

In April 2021 a ‘buy-out’ program will be put in place to acquire residential properties affected by the LERZ eruption^15^, up to a price of $230,000. A powerful proviso in the program prohibits use of the grants for house construction and infrastructure in lava hazard zones 1 and 2. Will this be sufficient incentive to encourage people to move to land that is outside of these high risk zones? Today, landowners face the difficult choice between the buy-out program and re-developing their existing properties with the certain, but low, risk of exposure in future LERZ eruptions. Affected residents fall into at least 3 different groups: those who lost their homes, those whose homes were isolated by the lava flows, and those living in parts of hazard zones 1 and 2 unaffected by this eruption. A solution in 2020 that satisfies all three groups is hard to achieve.

While there is continued uncertainty as to exactly when and where the next eruption will occur, the risk associated with land, lava, and development on Kīlauea remains today. Any permanent risk reduction requires a long-term plan to, for example, phase out residential development in lava hazard zones 1 and 2 over, say, 50 years. Will there be the changes in attitude and reaction driven by the scale of destruction in 2018 that permit such action, or will we turn their back on this large eruption, as has happened during every past event?
